# *In Vitro* and *In Vivo* Anti-AChE and Antioxidative Effects of *Schisandra chinensis* Extract: A Potential Candidate for Alzheimer's Disease

**DOI:** 10.1155/2020/2804849

**Published:** 2020-02-20

**Authors:** Xinmeng Song, Tiejie Wang, Linxiu Guo, Yibao Jin, Jue Wang, Guo Yin, Kun Jiang, Lijun Wang, Hongrong Huang, Long Zeng

**Affiliations:** Shenzhen Key Laboratory of Drug Quality Standard Research, Shenzhen Institute for Drug Control, Shenzhen, China

## Abstract

Acetylcholinesterase (AChE) inhibition and antioxidants are two common strategies for the treatment in the early stage of Alzheimer's Disease (AD). In this study, extracts from nine traditional Chinese medical (TCM) herbs were tested for anti-AChE activity by Ellman's microplate assay and cytotoxicity by CCK-8. Based on its excellent AChE inhibition effect and its lowest cytotoxicity, *Schisandra chinensis* (SC) extract was selected to do the mechanism research. SC extract protected pheochromocytoma (PC12) cells against H_2_O_2_-induced toxicity by improving the cell survival rate in a dose-dependent manner. And it also showed significant free radical (DPPH) scavenging activities, ferric reducing antioxidant power (FRAP), and 2,2′-Azino-bis-(3-ethylbenzothiazoline-6-sulfonic acid) (ABTS) radical scavenging. To confirm these results, the scopolamine-induced mice models were utilized in this study. Compared with the positive drug (piracetam), SC could also exhibit similar effects to alleviate the mice's cognitive deficits. Moreover, in the mice brain samples, the AChE activity and malondialdehyde (MDA) levels of SC-treatment group both showed a reverse as compared to model group. Taken together, these results all suggested that SC extract may be a potential therapeutic candidate for AD.

## 1. Introduction

With the rapid increase of population aging in recent years, senile dementia has become one of the most important public health problems in the world [1, 2]. Alzheimer's disease (AD), as the most common type of dementia, accounts for up to 70% of all cases of dementia. It is associated with substantial healthcare challenges, leading to a serious economic burden on both families and societies [3, 4]. Recently, AD has been verified to be a chronic neurodegenerative disease characterized by progressive memory loss and cognitive function impairment which is caused by the accumulation of senile plaques and neurofibrillary tangles in the brain [5, 6].

In current clinical practice, the drugs of acetylcholinesterase inhibitors and N-methyl-D-aspartate (NMDA) receptor antagonists are the mainstay for the treatment of AD [7, 8]. Reductions in the activity of the cholinergic neuron were always been tested in AD patients, and thus one of the important strategies for the therapy is to reduce the degradation of acetylcholine in neuromuscular junctions. Acetylcholinesterase inhibitors such as galantamine and donepezil are employed to increase the concentration of acetylcholine in the brain and combat the loss of acetylcholine caused by the death of cholinergic neurons [9, 10]. On the other hand, NMDA antagonist could reduce the glutamic acid-induced neurotoxicity by the inhibition of activity of NMDA to improve the cognitive function [11]. However, none of these therapies has significant effects on curing AD [12]. Therefore, it is indispensable to develop more novel and effective drugs for AD.

Traditional Chinese medicine (TCM) has already been used and even practiced in clinic over two thousand years in China. They could serve as an abundant drug library for screening the potential drugs to treat AD [13, 14]. Based on the theory of TCM, kidney stores essence, essence generates marrow, and the brain is the sea of marrow [15]. The deficiency in the sea of marrow can cause mental deficiency and loss of memory [15, 16]. Therefore, the principle of traditional Chinese medical therapy is to boost Qi, move the blood, regulate the spirit, and then sharpen cognition. In present study, nine Chinese herbs were selected to evaluate the potential for treatment of AD because they were most frequently used to improve the kidney function in books of TCM [17–23].

Among the representative hypotheses of mechanism associated with AD, the effects of AChE inhibition and oxidative stress reduction were focused on in this study [24]. As a result, the *Schisandra chinensis* (SC) was selected among the above nine Chinese herbs because of its potent inhibition effect on AChE and low cytotoxicity. For the possible bioactive components isolated from SC, the lignans like Schizarin A, B, C, D, and E were reported to exhibit antioxidant activity and some other lignans like gomisin A, C, D, and G along with gallotannins showed excellent inhibitory effects for AChE [25–28]. Hence, the potential mechanisms of SC were focused on the antioxidative activity and AChE inhibitory effects and on further exploration. Moreover, the effects of SC extract were also validated in scopolamine-induced animal model. Taken together, our work suggested that SC could be a potential candidate for the development of drugs for AD treatment.

## 2. Materials and Methods

### 2.1. Chemicals and Reagents

Dimethylsulfoxide (DMSO, anhydrous, ≥99.9%, 276855-100ML), acetylthiocholine iodide (ATCI, ≥98%, A5751-1G), AChE (*Electrophorus electricus*, Type VI-S, lyophilized powder, 200–1,000 units/mg protein, C3389-2KU), and 5,5′-Dithiobis (2-nitrobenzoic acid) (DTNB, ≥98%, D8130-1G) were purchased from Sigma-Aldrich (Sigma-Aldrich China, Inc.). 2,2′-Diphenyl-1-picryl hydrazyl radical (DPPH) kit, Ferric Reducing Ability of Plasma (FRAP) kit, 2,2′-Azino-bis (3-ethylbenzthiazoline-6-sulfonic acid (ABTS) kit, Lipid Peroxidation MDA Assay kit, Cell Counting kit-8, and DCF-DA kit were purchased from Beyotime Biotechnology. PBS, RPMI-1640 medium, fetal bovine serum, and Penicillin-Streptomycin were purchased from Gibco (ThermoFisher Scientific, USA). Scopolamine (No. 10013) was bought from National Institutes for Food and Drug Control. Piracetam Tablets were bought from Huanan Pharmaceutical Industry Group (H44020779).

### 2.2. Preparation of TCM Extracts

All nine TCM herbs (*Gastrodia elata, Schisandra chinensis, Dioscorea nipponica, Salvia miltiorrhiza, Polygonum multiflorum, Tribulus terrestris, Polygala tenuifolia, Amomum villosum,* and *Uncaria rhynchophylla*) were purchased from Tong Ren Tang (Beijing). Powdered herbs (5 g) were grinded and extracted with both distilled water (70 mL) and ethanol (70 mL) under reflux for three times, respectively. The extract was collected, filtered, and concentrated to a volume of 10 ml. After freeze-drying, the powder was then solubilized in DMSO (10 g/mL) stock to be used in cell culture studies.

### 2.3. Determination of AChE Inhibitory Activity

In present study, the AChE activities of the above nine TCM extracts were determined by the Ellman's microplate assay with a slight modification based on the reference reported in [29]. To each well of 96-well plate, 40 *μ*L of 50 mM Tris-HCl buffer (pH 8.0), 20 *μ*L of TCM extracts at the concentration of 20–5000 *μ*g/ml (20 *μ*L of PBS was used as 0 *μ*g/ml), 20 *μ*L of AChE solution (0.5 U/ml), and 100 *μ*L of DTNB solution (5 mM) were added in sequence. Huperzine A was prepared in the same buffer and used as the positive control. The obtained mixture was incubated at room temperature for 10 min. Then, the reactions were started by adding 20 *μ*L of ATCI solution (15 mM) to the mixture, and the absorbance was read at 412 nm every 30 s for 20 min. For blank experiment, only the same volume of Tris-HCl buffer was used instead of AChE solution. All conditions of the experiment were done in triplicate for the statistical analysis, and the percentage of AChE activity was calculated by the normalization to the group of 0 *μ*g/ml TCM extract. The IC_50_ value was calculated as the concentration of TCM extract that inhibited the AChE activity by 50%.

### 2.4. Cell Culture and Cell Viability Assay

Rat pheochromocytoma PC12 cells (Shanghai Institute of Biochemistry and Cell Biology, CAS, China) were cultured in RPMI 1640 medium supplemented with 10% FBS and maintained at 37°C in a humidified atmosphere of 5% CO_2_. PC12 cells which reached 80% confluence were used in the following in vitro experiments. The cell viability was detected by Cell Counting kit-8 (CCK-8). Briefly, 5000 PC12 cells were seeded in 96-well plates and allowed to adhere overnight. Then the cells were exposed to the nine TCM extracts at the doses of 0.02–10 mg/ml with a serious dilution. After 48 h treatment, 10 *μ*L of CCK-8 solution was added to each well and incubated for 2 h at 37°C; then the absorbance was directly measured at 450 nm. The cell viability was obtained by the normalization of each well to the vehicle control group using absorbance. And the cell viability versus TCM extract concentration was plotted and used for IC_50_ determination. The IC_50_ value was calculated as the concentration of TCM extract that induced the cell viabilities by 50%. Each condition was done in triplicate and the data were shown as mean ± SD.

### 2.5. Determination of Antioxidant Activities of SC Extracts

#### 2.5.1. Preparation of SC Extracts

The extracts of SC were prepared by water, 30% alcohol, 60% alcohol, and 90% alcohol, respectively, using the methods described in the above section.

#### 2.5.2. DPPH Assay

Antioxidant activities of different SC extracts were assessed through DPPH free radical assay in a microplate method based on the description in reference to a slight modification [30]. To each well, 190 *μ*L of SC extracts (1 mg/ml was used in this experiment) was added and reacted with 10 *μ*L of DPPH ethanol solution (1 mM) for one hour incubation at dark. The absorbance was then measured at 517 nm and used to evaluate its reducing capacity. The mixture of PBS solution and DPPH solution in the same volume was prepared for control group, Trolox was applied as the positive control, and a series of concentrations of 0.15, 0.3, 0.6, 0.9, 1.2, and 1.5 mM were used for standard curve. Each condition was done in triplicate, and the data were shown as mean ± SD. Results of SC extract's antioxidant capacity were expressed as mM Trolox equivalents.

#### 2.5.3. FRAP Assay

The ferric reducing ability of SC extracts was examined according to the description in reference to a slight modification [31]. The reaction mixture was composed with 180 *μ*L of FRAP working solution and 5 *μ*L of sample solution (PBS, SC extract, or positive control). Then the absorbance was read at 595 nm after incubation at 37°C for 3–5 min. Ferrous sulfate (FeSO_4_) was used as positive control, and the concentrations of 0.15, 0.3, 0.6, 0.9, 1.2, and 1.5 mM were applied for standard curve establishment. Results of SC extract's reducing capacity were expressed as mM Fe(II) equivalents.

#### 2.5.4. ABTS Assay

The ABTS radical scavenging activity of SC aqueous extract was assessed following a modified procedure developed by Re et al. [32]. Each well of 96-well plate was filled with 20 *μ*L of catalase working solution (1/500 from kit), 10 *μ*L of SC extract (1 mg/ml, using PBS as control and Trolox as positive control), and 170 *μ*L of ABTS working solution. For Trolox standard solutions, the concentrations were set as 0.15, 0.3, 0.6, 0.9, 1.2, and 1.5 mM. After mild mixing and incubation of 6 min at room temperature, the absorbance was measured at 415 nm. Each condition was done in triplicate, and the data were shown as mean ± SD. Results of SC extract's radical scavenging activity were expressed as mM Trolox equivalents.

### 2.6. Determination of Intracellular Reactive Oxygen Species Levels

The intracellular reactive oxygen species (ROS) scavenging capacity of SC extracts was evaluated using a DCFH-DA fluorescent probe based on the kit manual with a slight modification. After the treatment with different conditions as indicated, cells were washed twice with PBS solution and then incubated with DCFH-DA staining solution (final concentration 10 *μ*M) for 20 min at 37°C. Then, the stained cells were washed twice with PBS, and the fluorescence intensity was measured by microplate reader (Ex/Em = 485 nm/490 nm). The fluorescence intensity of control group was normalized as 1.

### 2.7. Animals

**Figure 1:**
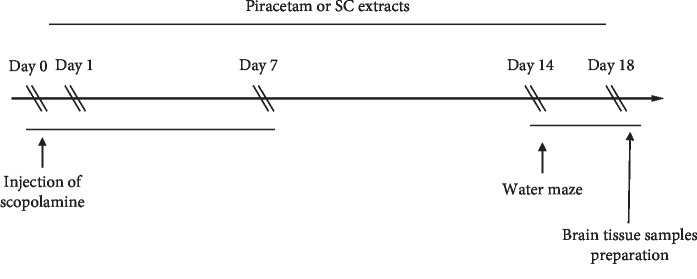
The schedule of animal experiments.

Forty KM mice (eight weeks old, 25–30 g) were purchased from Guangdong Medical Science Experiment Center (Approval No. SCXK-2018-0002) and randomly divided into four groups: vehicle group, AD-model group, piracetam (positive drug) group, and SC extract group, respectively. From day zero, mice except vehicle group received intravenous (*i.v*.) injections (5 mg/kg) of scopolamine once per two days for seven days while the mice from vehicle group were given equal volume of saline solution. As for drug treatment, from day one to the end of the experiment, mice from piracetam group and SC extract group were orally administrated 4 mg/kg piracetam and 10 mg/kg of SC extract, respectively. On the 14th day, the five-consecutive-day water maze experiments were used to examine the memory ability. The schedule of animal experiments is shown in Figure 1.

### 2.8. Morris Water Maze

For evaluation of mice memory ability, the Morris water maze test was conducted as described in [33]. Briefly, a black plastic circular pool (diameter, 1.0 m; depth, 55 cm) was filled with a depth of 30 cm water at room temperature. The pool was equally divided into four quadrants, and a clear circular platform (10 cm in diameter) was placed and submerged 2 cm below the surface of the water in the northwest quadrant of the pool. The mice behavior was recorded and analyzed by the SuperMaze video system (Shanghai Xinruan Information Technology Co. Ltd, Shanghai, China).

Before starting the experiment, all mice were firstly trained to find the circular platform for two days with a 24 hour interval. For the formal experiment, it was conducted after the training section, mice were place into the pool in the random quadrants and allowed to reach the platform. The escape latency was recorded as the time for a mouse finding and climbing the platform. A help was given to those mice failing to find the platform, keeping them on platform for 30 seconds. On the last day of assay, the platform was removed from the pool, and mice were put into the pool at the opposite quadrant of platform and allowed to swim freely for 60 s. The time spent and the routine of movement were recorded and calculated for the evaluation of performance.

### 2.9. Brain Tissue Preparation

After the last water maze experiment on the 18th day, all mice were executed and the whole brain samples were harvested for further study use. The obtained brain samples were washed by PBS and weighted for normalization. An appropriate volume of lysis buffer (1 ml lysis buffer for 0.1 g brain sample) was added for homogenization in the Dounce homogenizer for 10 times. Spinning was performed at 2,000 g for 5 min, and the supernatant was transferred to a new tube for the next biochemical analysis.

### 2.10. Measurement of AChE, SOD, FRAP, and Lipid Peroxidation in Brain Sample

The activities of AChE and FRAP of rat's brain sample were measured using Ellman assay as described in the SC extract evaluation. The other antioxidative enzyme activities like superoxide dismutase (SOD) and lipid peroxidation were measured by means of the assay kits manual from Beyotime Biotechnology (Shanghai, China).

### 2.11. Statistical Analysis

Data were expressed as mean ± standard deviation (SD). Statistical analysis between multiple groups was performed using ANOVA, followed by Tukey-Kramer tests. And differences between two groups were determined by Student's *t*-tests. Statistical significance was accepted with a two-tailed *p* value under 0.05 (*α* = 0.05). All data were analyzed and plotted by GraphPad Prism (version 6.0, CA, the USA).

## 3. Results

### 3.1. Ellman's Microplate Assay for Screening the AChE Inhibitory Activities

**Figure 2:**
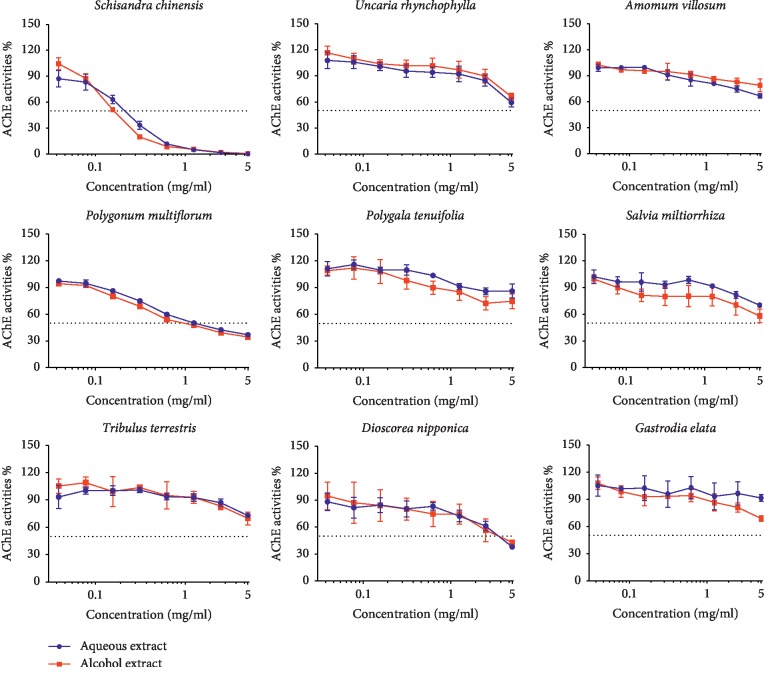
The evaluation of AChE activity of nine Chinese herbs by Ellman assay with water extraction (blue line) and alcohol extraction (red line).

A total of nine Chinese herb medicines were collected and extracted with water and alcohol, respectively, to yield 18 extracts. The AChE inhibitory activities of these 18 extracts were tested for their AChE inhibitory activities by Ellman's assay using microplate assay and the results are shown in Table 1 and Figure 2. It was found that the aqueous extract and alcohol extract exerted similar AChE inhibitory activities, and the extracts from *Dioscorea nipponica*, *Polygonum multiflorum*, and SC showed the strongest activities, while the rest of the extracts showed no or very weak AChE inhibitory activities. For these three extracts, their IC_50_ (the concentration at inhibition of 50% of AChE activity) values were around 4.65 mg/ml, 1.23 mg/ml, and 0.27 mg/ml, respectively.

### 3.2. The Cytotoxicity of the Three Selected Extracts

**Figure 3:**
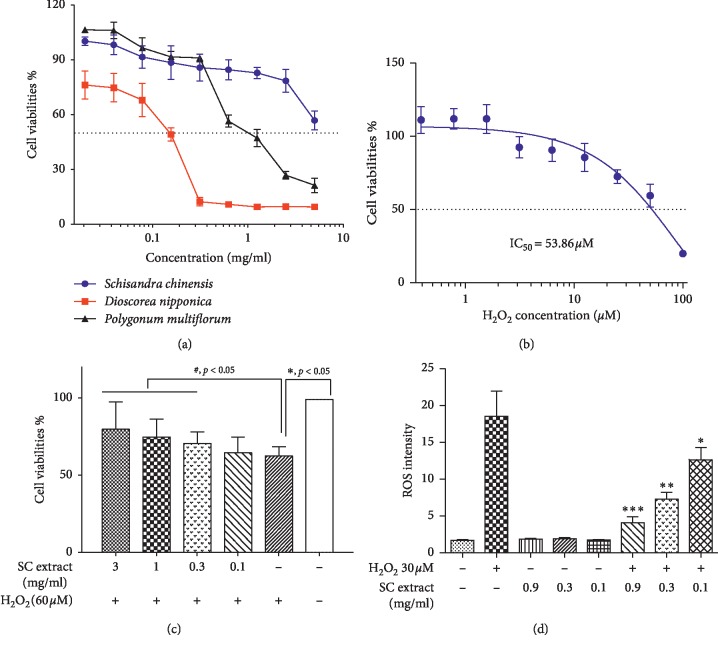
The cytotoxicity test and the neuroprotective effect of SC extract. (a) The CCK-8 assay of the selected three herbs against PC12 cells; (b) the cytotoxicity of H_2_O_2_ against PC12 cells; (c) the neuroprotective effect of SC extract against H_2_O_2_-induced oxidative damage. Values are mean ± SD (*n* = 3). ^∗^*p* < 0.05 compared with control group, ^#^*p* < 0.05 compared with H_2_O_2_ group. (d) The scavenging intracellular ROS effect of SC extract in a dose-dependent manner. Values are mean ± SD (*n* = 3). ^∗^*p* < 0.05, ^∗∗^*p* < 0 .01, ^∗∗∗^*p* < 0.001, compared with control group.

The cytotoxicity of the three activated extracts was evaluated against the PC12 cells using CCK-8. As shown in Figure 3(a), the highest cytotoxicity was observed for extract of *Dioscorea nipponica* with 50% inhibition at a concentration >5 mg/ml, followed by the aqueous extract of *Polygonum multiflorum* and the aqueous extract of SC, which showed IC_50_ values (the concentration at inhibition of 50% of cell viability) of 1.38 mg/ml and 0.12 mg/ml, respectively. By combination of the inhibitory effects and cytotoxicity results, SC showed the best AChE inhibition effect and the lowest cytotoxicity against neuron cells; hence SC was considered as the best herb for the further research.

### 3.3. The Neuroprotective Effect of SC Extract

The preservative effect of SC extract was examined using the PC12 cell oxidative damage models induced by H_2_O_2_. As shown in Figure 3(b), through the test of the cytotoxicity of H_2_O_2_ against PC12 cell lines, 60 *μ*M of H_2_O_2_ was used to induce the cell oxidative damage. Consistent with our expectation, the cell viability of the PC12 cells exposed to SC extract decreased the H_2_O_2_-induced cytotoxicity in a dose-dependent manner (Figure 3(c)). When the SC extract is over 0.3 mg/ml, the neuroprotective effects showed a statistical difference compared to H_2_O_2_-induced model group (*p* < 0.05).

### 3.4. The Scavenging Intracellular ROS Effect of SC Extract

As for the neuroprotective effects of SC extract shown in H_2_O_2_-induced models, we supposed that SC extract could remove the reactive oxygen species. Therefore, the fluorescent probe of DCFH-DA was used to determine the intracellular ROS levels. As is shown in Figure 3(d), the ROS level was increased up to approximately 600% of control by 30 *μ*M of H_2_O_2_-treatment, and the increased ROS was effectively inhibited by SC extract in a concentration-dependent manner.

### 3.5. The *In Vitro* Antioxidative Effect of SC Extract

**Figure 4:**
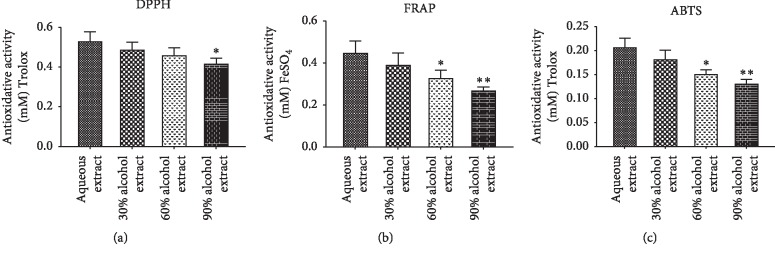
The *in vitro* antioxidative effect of different SC extracts determined by DPPH (a), FRAP (b), and ABTS (c). All values are means ± SD (*n* = 3). ^∗^*p* < 0.05, ^∗∗^*p* < 0.01, compared with aqueous extract group.

In the present study, three analysis methods, namely, DPPH, FRAP, and ABTS, were applied to evaluate *the in vitro* antioxidant activity of SC extract. As shown in Figure 4(a), all SC extracts (1 mg/ml) possessed effective activity to scavenge DPPH radical. The scavenging activity of aqueous SC extract was the strongest scavenging capacity among four extract methods. Its activity was equal to 0.533 mM Trolox. For ferric reducing antioxidant power (FRAP) assay, a significant reducing activity was shown in Figure 4(b), and the aqueous extraction of SC also exhibited the strongest activity equal to 0.452 mM FeSO_4_. Similarly, through the ABTS assay, SC aqueous extract exhibited excellent antioxidative ability compared to the positive control Trolox (0.208 mM Trolox for 1 mg/ml extract) (Figure 4(c)).

### 3.6. Effects of SC Extract on Morris Water Maze Test

**Figure 5:**
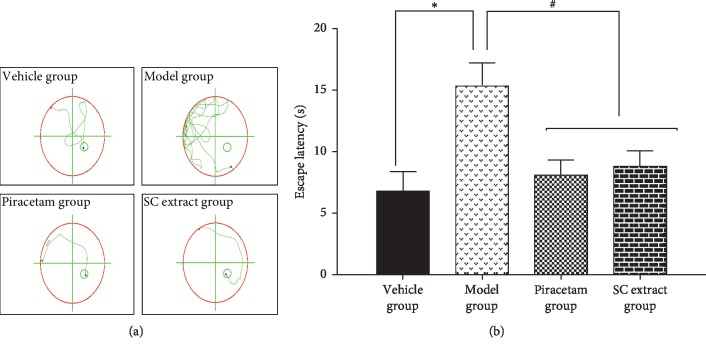
Effects of SC extract on search strategy which was recorded by swimming movement focus (a) and escape latency (b) of the Morris water maze test. Values are means ± SD (*n* = 10). ^∗^*p* < 0.05 compared with control group; ^#^*p* < 0.05 compared with model group.

In order to evaluate the potential protective effects of SC extract *in vivo*, the Morris water maze assay was applied and used to test whether SC extract could improve the cognitive impairment on mice AD models induced by scopolamine. The spent time to find the position of removed platform, named escape latency, and the swimming movement focus for finding the invisible position were used to measure the performance of cognitive ability. The longer escape latency time and the more complicated swimming routes mean lower memory ability. As shown in Figure 5, the group induced by scopolamine showed a significant delay of the escape latency time compared with the vehicle group during the water maze tests, suggesting that the model group works. After the treatment of piracetam, the effect of scopolamine on escape latency was ameliorated as expected. Moreover, SC extract also significantly decreased the escape latency time of finding the platform on days 15, 16, and 17. Similarly, from the swimming routers (Figure 5(a)), the mice with memory deficit induced by scopolamine showed a rambling direction to find the platform after the treatment with SC extract, which suggested that the mice could find the platform more quickly, as piracetam did.

### 3.7. Effect of SC Extract on the Levels of AChE, SOD, and Lipid Peroxidation in Brain Sample

**Figure 6:**
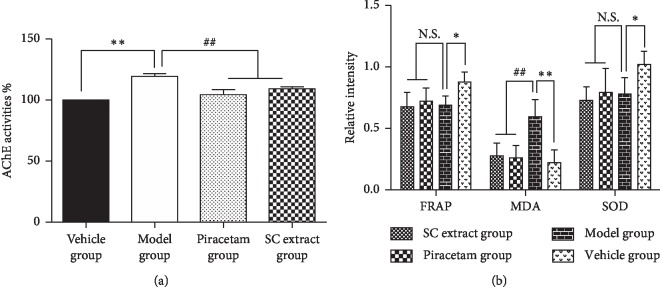
Effect of SC extract treatment on brain AChE and antioxidant enzymes activities (*n* = 10). (a) AChE; (b) FRAP, SOD, and MDA. Data are presented as mean ± SD. ^∗^*p* < 0.05 and *p* < 0.01*vs* vehicle group; ^#^*p* < 0.05 and ^##^*p* < 0.01*vs* scopolamine model group.

In order to confirm the AChE inhibition effect of SC extract *in vivo*, after the water maze test, all the brain samples were collected and homogenized to determine the AChE level by Ellman assay. As shown in Figure 6(a), the AChE activity was increased in model group and decreased to a level close to the normal group by the treatment of piracetam or SC extract. It was suggested that the SC extract possessed AChE inhibition effect both *in vivo* and *in vitro*.

Recently, the oxidative stress was shown to be closely related to neurodegenerative diseases. The overexpressed reactive oxygen species always cause radical damage to the neuron cells. Antioxidant reagents nowadays are considered as promising therapeutics against AD. Therefore, the three kinds of representative ROS, namely, enzymatic antioxidants (SOD), reducing power antioxidants, and lipid peroxidation (based on malondialdehyde, MDA) in the brain tissue were determined. As illustrated in Figure 6(b), in the model groups, the levels of SOD and FRAP in the brain tissue both showed an abnormal decrease rather than increase comparing with the vehicle group (*p* < 0.05). And after the treatment of piracetam or SC extract, the levels also did not show any reverse (*p* > 0.05). However, the MDA levels in mice brains showed a remarkable increase in 227.7% (*p* < 0.01) by scopolamine compared to the vehicle group, whereas they were significantly attenuated by piracetam and SC extract (*p* < 0.01). The MDA in brain was produced by the reaction of lipids with the oxidative species so that it was usually used for the measurement of oxidative stress. From the results, it is indicated that the decrease of oxidative stress by SC extract might play an important role with its anti-AD effect.

## 4. Discussion

Although a number of previous studies have been performed to elucidate the neuropathology molecular mechanisms of AD, only a few available therapies were used until now [34]. Moreover, the existing drugs for AD treatment were only used for the mild to moderate AD. Despite this, since 2004, there are no more drugs approved by Food and Drug Administration (FDA) for the treatment of AD. Hence, discovery of new effective drugs has become an active demand based on the situation of dramatic increase in AD cases in recent years.

SC is frequently used in China, Korea, and Japan as a tonic for kidney and a treatment for alleviating various symptoms of cognitive deficits and facilitating learning and memory for thousands of years [35]. Recent experience from clinic and laboratory supported the notion that SC was safe and effective in improving cognitive function and reversing cycloheximide-induced amnesia in rats [36]. And also, lignans like Schizarin A, B, C, D, and E were reported to exhibit the antioxidant activity, and some other lignans like gomisin A, C, D, and G along with gallotannins showed excellent inhibitory effects for AChE in several compounds [25–28]. However, as for the SC extract, the exact role for the treatment of AD has not been investigated yet.

In this study, SC was selected from nine frequently used Chinese herbs for treatment of AD based on the excellent *in vitro* inhibition activity of AChE along with the lowest cytotoxicity (Figure 2). We further tried to elucidate the underlying mechanisms. Because of the neuroprotective effect of SC extract against H_2_O_2_-induced oxidative damage (Figure 3), the antioxidative role of SC extract was focused on in our study. The oxidative stress was always found to be associated with the neurodegenerative diseases, and the antioxidants possess the potential to neutralize this oxidative damage so that it can help to prevent and positively improve certain aging diseases. [37, 38]. We then continued the assessment of the *in vitro* and *in vivo* antioxidant and neuroprotective activities of the SC extract. In PC12 cell culture, a significant protective effect improving cell survival percentage was shown after the treatment of SC extract against H_2_O_2_-induced toxicity in a dose-dependent manner. And it also showed excellent free radical (DPPH) scavenging activities, ferric reducing antioxidant power (FRAP), and ABTS radical scavenging (Figure 4). It was indicated that the antioxidative effect may be one of the major contributions of the SC extract.

Moreover, the anti-AD effect of SC extract was confirmed on the mouse model. Piracetam, a classical drug approved by FDA, was used as the positive control. Morris water maze was used to test the effect of SC extract treatment in behavior test. Scopolamine, a tropane alkaloid drug, was used to establish the AD mouse model as a muscarinic receptor antagonist since it can induce anterograde memory impairment, particularly short-term memory and learning acquisition [39]. As a result, the model was well established and verified by the reduction of the spontaneous alternation behavior in Morris water maze test and the increase of the escape latency that was observed after scopolamine treatment. After the treatment of SC extract or piracetam, we found that SC extract significantly ameliorated the cognitive impairment induced by scopolamine in a level similar to piracetam (Figure 5). Importantly, for the *in vivo* brain samples, the AChE activity also showed a decrease by the treatment of SC extract, and the MDA level was reversed compared with the model group (Figure 6). Taken together, all these results suggested that SC extract showed inhibition of AChE and antioxidative effect both *in vitro* and *in vivo* and might be useful for the AD treatment.

## 5. Conclusion

In summary, the data in this study suggested that the SC extract has the potential to reduce AChE activity and relieve the oxidative damage both *in vivo* and *in vitro*. Through the assays in PC12 cells and the AD mouse model induced by scopolamine, SC extract exhibited potent neuroprotective effects linked to antioxidative mechanisms. Moreover, based on the determination activity of AChE, the effects of SC may be partly attributed to its potential to inhibit the activity of AChE. Therefore, SC extract could serve as a multifunctional therapeutic remedy for the treatment of AD. The treatment with SC extract was potentially an effective and simple strategy to improve learning and memory abilities in the elderly and may be a feasible method to prevent the development of AD.

## Figures and Tables

**Table 1:** The IC_50_ values of aqueous and alcohol extracts for AChE inhibition assay.

	Aqueous extract (IC_50_, mg/ml)	Alcohol extract (IC_50_, mg/ml)
*Gastrodia elata*	>5	>5
*Schisandra chinensis (SC)*	0.22 ± 0.07	0.16 ± 0.06
*Dioscorea nipponica*	3.54 ± 0.22	3.54 ± 0.38
*Polygonum multiflorum*	1.26 ± 0.09	0.94 ± 0.06
*Tribulus terrestris*	>5	>5
*Salvia miltiorrhiza*	>5	>5
*Polygala tenuifolia*	>5	>5
*Amomum villosum*	>5	>5
*Uncaria rhynchophylla*	>5	>5

## Data Availability

The data used to support the findings of this study are included within the article.
